# Molecular Interplays Between Cell Invasion and Radioresistance That Lead to Poor Prognosis in Head-Neck Cancer

**DOI:** 10.3389/fonc.2021.681717

**Published:** 2021-07-09

**Authors:** Guo-Rung You, Joseph T. Chang, Yan-Liang Li, Yin-Ju Chen, Yu-Chen Huang, Kang-Hsing Fan, Yen-Chao Chen, Chung-Jan Kang, Ann-Joy Cheng

**Affiliations:** ^1^ Department of Medical Biotechnology and Laboratory Science, College of Medicine, Chang Gung University, Taoyuan, Taiwan; ^2^ Department of Radiation Oncology, Chang Gung Memorial Hospital-Linkou, Taoyuan, Taiwan; ^3^ School of Medicine, College of Medicine, Chang Gung University, Taoyuan, Taiwan; ^4^ Graduate Institute of Biomedical Materials and Tissue Engineering, College of Biomedical Engineering, Taipei Medical University, Taipei, Taiwan; ^5^ International Ph.D. Program in Biomedical Engineering, College of Biomedical Engineering, Taipei Medical University, Taipei, Taiwan; ^6^ Department of Oral Maxillofacial Surgery, Chang Gung Memorial Hospital-Linkou, Taoyuan, Taiwan; ^7^ Department of Radiation Oncology, New Taipei Municipal TuCheng Hospital, New Taipei City, Taiwan; ^8^ Department of Medical Imaging and Radiological Sciences, College of Medicine, Chang Gung University, Taoyuan, Taiwan; ^9^ Department of Radiation Oncology, Chang Gung Memorial Hospital-Keelung, Keelung, Taiwan; ^10^ Department of Otorhinolaryngology, Chang Gung Memorial Hospital-LinKou, Taoyuan, Taiwan; ^11^ Graduate Institute of Biomedical Sciences, College of Medicine, Chang Gung University, Taoyuan, Taiwan

**Keywords:** head-neck cancer, radioresistance, cell invasion, prognosis, signaling pathway, ITGA6 molecule

## Abstract

**Background:**

Cancer metastasis and recurrence after radiotherapy are the significant causes of poor prognosis in head-neck cancer (HNC). Clinically, it is commonly found that patients with either condition may accompany the outcome of the other. We hypothesized that HNC cells might exhibit a cross-phenotypic attribute between cell invasion and radioresistance. To discover effective biomarkers for the intervention of aggressive cancer at one time, the potential molecules that interplay between these two phenotypes were investigated.

**Materials and Methods:**

Three isogenic HNC cell sublines with high invasion or radioresistance properties were established. Transcriptomic and bioinformatic methods were used to globally assess the phenotypic-specific genes, functional pathways, and co-regulatory hub molecules. The associations of gene expressions with patient survival were analyzed by Kaplan-Meier plotter, a web-based tool, using the HNSCC dataset (n=500). The molecular and cellular techniques, including RT-qPCR, flow cytometry, cell invasion assay, and clonogenic survival assay, were applied.

**Results:**

The phenotypic crosstalk between cell invasion and radioresistance was validated, as shown by the existence of mutual properties in each HNC subline. A total of 695 genes was identified in associations with these two phenotypes, including 349 upregulated and 346 downregulated in HNC cells. The focal adhesion mechanism showed the most significant pathway to co-regulate these functions. In the analysis of 20 up-regulatory genes, a general portrait of correlative expression was found between these phenotypic cells (r=0.513, p=0.021), and nine molecules exhibited significant associations with poor prognosis in HNC patients (HR>1, p<0.050). Three hub genes were identified (ITGA6, TGFB1, and NDRG1) that represented a signature of interplayed molecules contributing to cell invasion, radioresistance and leading to poor prognosis. The ITGA6 was demonstrated as a prominent biomarker. The expression of ITGA6 correlated with the levels of several extracellular and apoptotic/anti-apoptotic molecules. Functionally, silencing ITGA6 suppressed cell migration, invasion, and attenuated radioresistance in HNC cells.

**Conclusions:**

A panel of interplay molecules was identified that contribute to cell invasion and radioresistance, leading to poor prognosis. These panel molecules, such as ITGA6, may serve as predictive markers of radioresistance, prognostic markers of metastasis, and molecular therapeutic targets for refractory HNC.

## Introduction

Head and neck cancer (HNC), including the oral cavity and oropharynx squamous cell carcinomas, is one of the ten leading cancers worldwide (1–3). This cancer usually occurs in the middle age male, at the high peak of life responsibility; it has a tremendous impact on family and society. The head and neck area is rich with lymphatic tissue; therefore, the bulky invasive tumors or lymph node metastases are often found in HNC (4, 5). In this context, gene products supporting invasion may be novel targets for manipulating the cancer behavior with consequences on treatment outcome. Several experimental approaches have been used to identify invasion-related genes in HNC, including comparing two sets of samples with different invasion capabilities (6) or comparing cancer cell lines with normal keratinocytes (7). However, a significant disadvantage of these approaches lies in the heterogeneity between samples. To reduce heterogeneity and obtain specific data on the gene expressions related to cancer invasiveness, we previously established several isogenic cancer cell sublines with highly invasive features derived from HNC cell lines (8, 9). The cDNA microarrays were performed to compare the differential transcriptome profile between invasive sublines and the parental cells. Heretical clustering analysis revealed 461 genes associated with cancer invasion, including 210 up-regulated and 251 down-regulated genes in the invasion sublines.

Radiation therapy is an indispensable part of the treatment of HNC. The identification of radioresistant molecules for further applications should contribute to a great improvement in treatment outcomes. Previously, microarrays have been used to compare gene expression profiles between parental and radiation-treated cancer cell lines in few cancers (10, 11). However, these cells were examined after a few hours or days of irradiation. The results of these gene alterations thus may represent the radiation response or induction molecules. To obtain a more thorough profile of molecules that may represent the intrinsic factor of radioresistance in HNC, we previously established several isogenic radioresistant sublines derived from HNC cancer cell lines (12, 13). The cDNA microarray database was established by comparing the gene expression profiles between radioresistant sublines and the parental cells. The heretical clustering analysis revealed 255 genes associated with radioresistance, including 155 up-regulated and 100 down-regulated in the radioresistant cells.

Clinically, the worse prognosis of HNC patients was often resulted from cancer metastasis or therapeutic resistance. Interesting, highly invasive cancers with nodal metastasis often accompany poor radiotherapeutic response (14, 15). Similarly, recurrent HNC patients with radioresistant cancers often have a higher metastasis rate (16, 17). From these clinical insights, we hypothesized that HNC cells might exhibit a cross-phenotypic attribute between cell invasion and radioresistance. We, therefore, employed the invasion- and radioresistant sublines as study models to examine the potential cross-regulatory mechanism. We determined a molecular panel and core pathways that may participate in the interplay of these two phenotypes through integrative analysis of the transcriptomic datasets. We further assessed the prognostic significance of these cross-regulatory molecules in HNC patients and concluded a panel of molecules facilitating worse survival. The cellular and molecular examinations demonstrated a hub gene, ITGA6, that played prominent roles in cellular invasion and radioresistance, leading to refractory cancer. Our study provides prognostic information, which may be further applied as molecular biomarkers and therapeutic targets for the treatment of refractory HNC.

## Materials and Methods

### Cell Lines and the Isogenic Sublines With Highly Invasive or Radioresistant Phenotypes

The HNC cell lines, OECM1, Detroit, Fadu and SAS were used in this study (8, 9). These cells were grown in MEM or RPMI 1640 medium supplemented with 10% fetal bovine serum. For establishment of highly invasive sublines, the Matrigel-invasion protocol was employed, and the selection cells were designed as the specific sublines (OECM-Inv, Detroit-Inv, Fadu-Inv) (8, 9). For establishment of radioresistant sublines, the serial irradiation method was used, and the survival cells were designated as RR sublines (OECM1-RR, FaDu-RR, Detroit-RR) (12, 13).

### Transcriptomic Profiling and Functional Pathways Associated With Cell Invasion and Radioresistance

The differential transcriptomes between HNC parental cells and the specific cell sublines were examined by using Affymetrix cDNA microarray (GeneChip Human Genome HG-U133A). The differentially expressed gene (DEG) was selected *via* ANOVA analytical method based on the criteria of average fold-change > 1.5 and P-value < 0.05 between parental and the subline cells. Hierarchical cluster analysis was applied to assess the similarity between sample groups. To determine the functional pathways associated with radioresistance and cell invasion, the DEGs identified in the microarrays were analyzed by using computational methods, the DAVID and the KEGG bioinformatic tools (https://david.ncifcrf.gov/) (13). Pathway enrichment analysis was applied to identify molecular pathways according to the KEGG database. Significantly enriched functional terms (adjusted p-values <0.05) for up- or down-regulated genes were reported.

### Clinical Assessment of Prognostic Significance in HNC Patients

The KM-Plotter online tool (http://kmplot.com/analysis) was also used to assess the prognostic significance of the cross-regulatory genes in HNC patients. The cohort of the TCGA-HNSC dataset was analyzed. This dataset contained 500 patients with head and neck squamous cell carcinoma and with prognostic information (18). High- and low-risk groups were classified using an optimization algorithm according to each gene expression level. The Kaplan-Meier analysis was performed to evaluate overall survival, and the log-rank test was used to calculate hazard ratios (HRs) and their 95% confidence intervals (CIs).

### Evaluation of the Differential Expression Genes by RT-qPCR Method

The differentially expressed levels of the genes between HNC parental cell lines, the RR sublines, and the Invasion sublines were evaluated using RT-qPCR method (9, 19). Briefly, the cDNA synthesis and qPCR were performed using the MiniOpticon™ real-time PCR detection system and SYBR Green Supermix reagents. Total of 20 genes were examined. The primers used in this study were listed in **Supplementary Table S3**.

### Construction of sh-ITGA6 Plasmid and Cellular Transfection

Construction of the short hairpin (sh)-ITGA6 plasmid, and the following transfection experiments were performed similarly as previously described (8, 20). The sense and antisense hairpin nucleotides complementary to ITGA6 mRNA were also generated and cloned into the pLKO.1 vector plasmid. The sequence for sh-ITGA6 is 5’-ATT- AAT- CTG- AAG- TTA- GAA- CA- CCT- TCT- TCT-AAC-TTC- AGA- TTA- AT-3’. The plasmids were transfected into cells using Lipofectamine 2000 reagent with Opti-MEM medium (Invitrogen, USA) according to the manufacturer’s instructions. After transfection, the Opti-MEM medium were replaced with fresh complete medium. Cellular clones that were stably transfected with sh-ITGA6 plasmid were selected using the neomycin antibiotic G418.

### Determination of Radiosensitivity by Clonogenic Survival Assay

Radiosensitivity was determined by clonogenic survival assay as previously described (12, 20). Briefly, cells were seeded into a 6-well cell culture plate for 8 hours. The cells were exposed to various dose of radiation (0 to 6 Gy) and then continuously cultured for 7-14 days to allow cell colony formation. The survival fraction was calculated as the number of colonies divided by the number of seeded cells times the plating efficiency.

### Determination of Cell Migration and Invasion Abilities

The cell migration ability was determined by using the *in vitro* wound-healing assay (8, 9). Briefly, cells were seeded in an ibidi^®^ culture insert (Applied BioPhysics, Inc. NY) on top of a 6-well plate. After 8 hr of incubation, the culture insert was detached to form a cell-free gap in a monolayer of cells. After changing to culture medium with 1% FCS, the cell migration status toward the gap area were photographed with a specific period time point. The cell invasion ability was evaluated by using the BioCoat Matrigel (Becton Dickinson Biosciences, Bedford, MA) and Millicell invasion chamber (Millipore Corporation, Bedford, MA) (8, 9). The Matrigel were fist coated onto the membrane of the Millicell upper chamber with a pore size of 8 μm in a 24-well plate. Cells in 1% FBS medium were seeded into the upper chamber. The lower chamber will contain 10% FBS in medium to trap invading cells. After a specific time point, the cells invading to the reverse side of the membrane were fixed, stained, and photographed.

### Evaluation of Cell Cycle Status by Flow Cytometry

The cell cycle status was determined by flow cytometry analysis, similarly as previously described (9, 19). Briefly, the cells were first synchronized to G0 phase by replacing the culture medium with serum-free medium. After 24 hr, cells in the exponential phase were collected and fixed with ice-cold 70% ethyl alcohol in PBS. Cells were then permeabilization with Triton X-100 solution, stained with propidium iodide solution, and analyzed by a FACScan flow cytometry (Becton Dickinson). The distribution of cell cycle phases was determined using Cell Quest Pro and ModiFit software.

### Measurement of Cellular Reactive Oxygen Species (ROS) Level

Intracellular ROS level were measured by the H2DCF-DA oxidation method (Invitrogen, Carlsbad, CA, USA) similarly as previously described (12, 20). Briefly, cells were grown on coverslip plates in HEPES buffer supplemented with H2DCF-DA reagent. The H2DCF-DA is a cell-permeable probe that is oxidized by intracellular ROS to generate fluorescent DCF. The green fluorescence of DCF was monitored by flow cytometric analysis (FACSCalibur, BD Biosciences, Franklin Lakes, NJ, USA).

### Statistical Analysis

The two-tailed unpaired Student’s t-test was used for the comparison of two variables between the means. All statistical analyses were conducted using a significance level of P < 0.05.

## Results

### Phenotypic Cross-Talk Between Cell Invasion and Radioresistance in HNC

**Figure 1:**
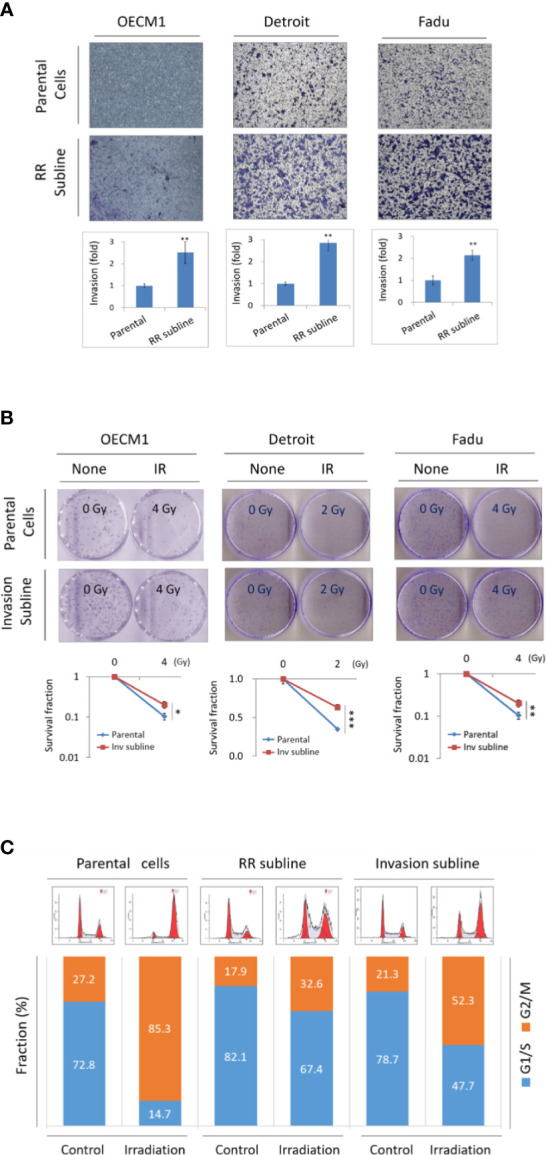
Phenotypic cross-talk between cell invasion and radioresistance in HNC. **(A)** Radioresistant sublines exhibited higher cell invasion ability compared to the parental cells, as determined by Matrigel invasion assay. Total of 3 HNC parental cell lines (OECM1, Detroit, and Fadu) and their radioresistance sublines (RR sublines) were examined. The numbers of cells that had invaded through the Matrigel to the reverse side were stained, photographed and quantified. **(B)** The invasion sublines showed statistically more resistant to irradiation compared to their parental cells, as determined by clonogenic survival assay. Total of 3 HNC parental cell lines (OECM1, Detroit, and Fadu) and their invasion sublines were examined. The colony survival fractions were determined after the cells were irradiated with 2 or 4 Gy. The experiments were performed for three times, and the similar results were obtained. The error bars shown in the relevant figures indicated the standard deviation of the three independent experiments. (*p < 0.05, **p < 0.01, ***p < 0.001, t-test). **(C)** Both Invasion and radioresistant sublines enrich cells at G1/S phase in response to irradiation. The OECM1 parental cell, the RR subline or the invasion subline were examined. Cells were then synchronized to the G0 phase by replacing the culture medium with serum-free medium. The cells were treated with a single dose of 6 Gy of irradiation and continuously cultured for 24 hours. In each sample, cell cycle distribution was determined by flow cytometry analysis.

Previously, we have established several high invasion sublines and radioresistance sublines derived from HNC cancer cell lines (8, 9, 12, 13). In this study, we determined whether these two phenotypes may possess a phenotypic cross-regulatory attribute. The cell invasion and radiosensitivity were determined by Matrigel invasion and clonogenic survival methods. First, we assessed the invasion ability in HNC parental cells and the radioresistant sublines (OEC-RR, Det-RR, and Fadu-RR) after confirming higher radioresistance in the RR cells. As shown in **Figure 1A**, the RR sublines possessed higher invasion ability than their parental cells, with the 2.1- to 2.9-fold increase in three RR sublines. We next examined the radiosensitivity in parental cells and the invasion sublines (OEC-Inv, Det-Inv, Fadu-Inv). As shown in **Figure 1B**, these invasion sublines exhibited higher resistance to irradiation by increasing 1.3- to 1.8-fold in these invasive sublines compared to the parental cells.

It has been reported that the cell cycle lying at the G1/S phase was more resistant to irradiation while G2/M is more sensitive (12, 21). We examined whether the RR- and invasion sublines may have the favorable cellular phase of G1/S in common when responsive to irradiation. The flow cytometry was performed to analyze the distribution of cell cycle status after 24 hr of radiation treatment. The results were shown in **Figure 1C**. Without irradiation, the cellular fractions at the G1/S phase were at a similar level as in parental cells, the RR sublines, or the invasion sublines (73%, 82%, and 79%, respectively). Upon irradiation, cells were transited from G1/S to the G2/M phase in general. However, the RR and invasion sublines exhibited more reluctance to this transition, as shown by higher G1/S fraction than the parental cells (15%, 67%, and 48%, respectively). The ratio of cellular fraction in G1/S verse G2/M increased approximately 3- and 2-folds respectively in the RR and invasion sublines. Thus, the RR and invasion sublines possessed a similar characteristic that being less sensitive to irradiation. These results suggested that HNC cells exhibited an attribute of phenotypic cross-talk between cell invasion and radioresistance.

### Functional Pathways in Cross-Regulation of Cell Invasion and Radioresistance in HNC

**Figure 2:**
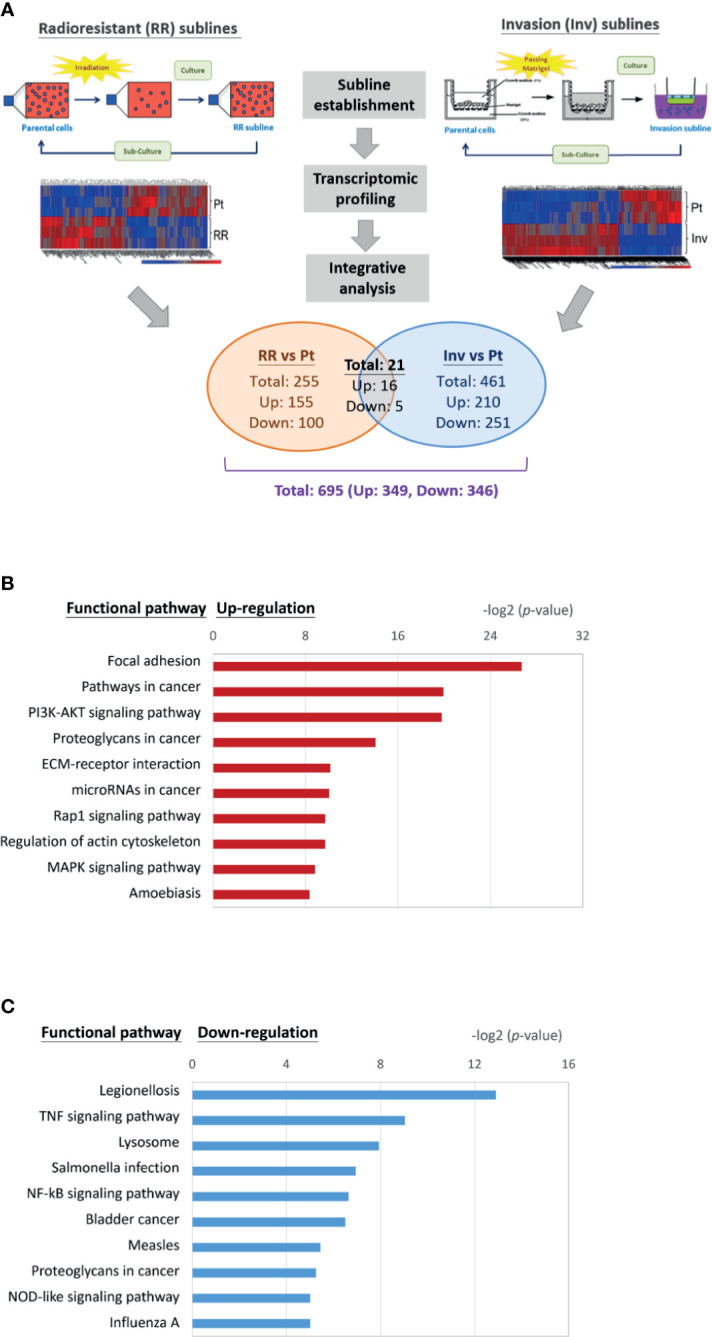
Transcriptomic profile and molecular pathways associated with cross-regulating function on cell invasion and radioresistance. **(A)** Conceptional design of the analytical strategy to investigate cross-regulatory genes for cell invasion and radioresistance. The profiles of differentially expressed genes (DEGs) were established after comparison of the transcriptomic datasets from three HNC cell lines (OECM1, Detroid, Fadu) and their sublines. **(B)** A list of the top 10 significant molecular pathways determined by DAVID enrichment analysis of the 349 up-regulatory genes. **(C)** A list of the top 10 significant molecular pathways determined by DAVID enrichment analysis of the 346 down-regulatory genes. Bar chart representing the classification of KEGG network. The enriched significance (p-value) values were negative base-2 log-transformed.

The functional pathways that may crossly regulate cell invasion and radioresistance were investigated. We integrated the transcriptomic datasets of the DEG profiles from three HNC cell lines and their respective sublines to obtain more comprehensive information. **Figure 2A** showed the conceptional design of this analytical strategy. We applied the bioinformatics software to identify common hub genes between multiple cell lines to comprise heterogeneous cancers. After combinational analysis of these datasets, there were only 21 genes differentially expressed in both phenotypes of the three HNC cell lines (**Supplementary Table S1**). The limited number of molecules may be due to intrinsic heterogeneity of genetic background within multiple cell lines. The DEGs in either phenotype of these three sublines were recruited to increase the dataset of potential cross-regulatory genes. A total of 695 genes were obtained, with 349 up-regulated and 346 down-regulated compared to the parental cells.

The up-regulated (349) or down-regulated (346) genes were imported to the KEGG suite for molecular network analysis. The top 10 pathways with either up or down-regulation were shown in **Figures 2B, C**. In the up-regulatory pathways, these molecules were enriched related to oncogenic function in general. The cell motility mechanism was most significant, as the regulation of focal adhesion, the association with extracellular matrix (ECM)-receptor interaction, and actin cytoskeleton regulation. The other molecular mechanisms participating in oncogenic signaling pathways were also apparent, as the PI3K-Akt, the Rap1 signaling, and the MAPK signaling (**Figure 2B**). In the down-regulatory pathways, these molecules were enriched most related to infectious diseases or immune/stress responses (**Figure 2C**). These included the conditions of legionellosis, the signaling pathways of TNF-regulatory, NF-kB, or NOD-like signaling. In all, these results indicate that the functional process participating in the interplay of cell invasion and radioresistance involves a wide range of molecular mechanisms, which may be required to maintain homeostasis in HNC cells. Note that focal adhesion regulation showed at the top-ranking among all pathways (P= 8.29E-27), indicating this mechanism’s prominence in the cross-regulatory function of cell invasion and radioresistance.

### Panel Molecules Correlative Up-Regulation in the Invasion and Radioresistant Cells

**Figure 3:**
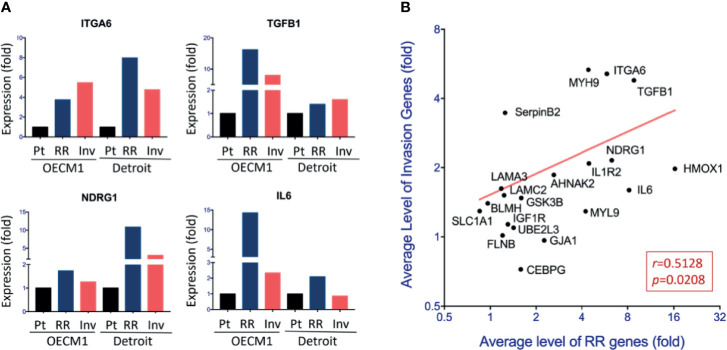
Panel molecules correlative up-regulation in the invasion and radioresistant cells. **(A)** Relative levels of gene expressions among the parental (Pt) cells, radioresistant (RR) subline, and invasion (Inv) subline of the OECM1 and Detroit cell lines, by using RT-qPCR method. The expression levels are shown by bars. **(B)** Correlative expressions of 20 genes between RR subline and Inv subline of HNC cells. For each gene, the average level of the fold changes compared to the parental cells from two cell lines (OECM1 and Detroit) was used.

We next parallelly investigated the gene expression levels in both sublines and their parental cells of the two HNC cell lines to validate the potential molecules that may crossly regulate cell invasion and radioresistance. A total of 20 genes were selected and subjected to RT-qPCR examination. These genes included the 16 up-regulations in the transcriptomic study’s phenotypes and the four related to the focal adhesion functional pathway. **Figure 3A** showed examples of the results, and **Supplementary Table S2** summarized all the data. Although various levels in differential cell sublines, many genes were elevated in both invasion and RR cells, including ITGA6, TGFB1, NDRG1, and IL6. The over-expression levels in these two phenotypic cells were plotted for each gene to assess a typical set of hub genes that may co-regulate cell invasion and radioresistance. We averaged the gene expression levels in two HNC cell lines to comprise heterogeneity between different cell lines. As shown in **Figure 3B**, these genes were correlatively expressed in the invasion and RR sublines (r=0.5128, p=0.0208). These results suggested a panel of genes that contribute to both phenotypes.

### Molecular Interplays Between Cell Invasion and Radioresistance That Led to Poor Prognosis in HNC Patients

**Figure 4:**
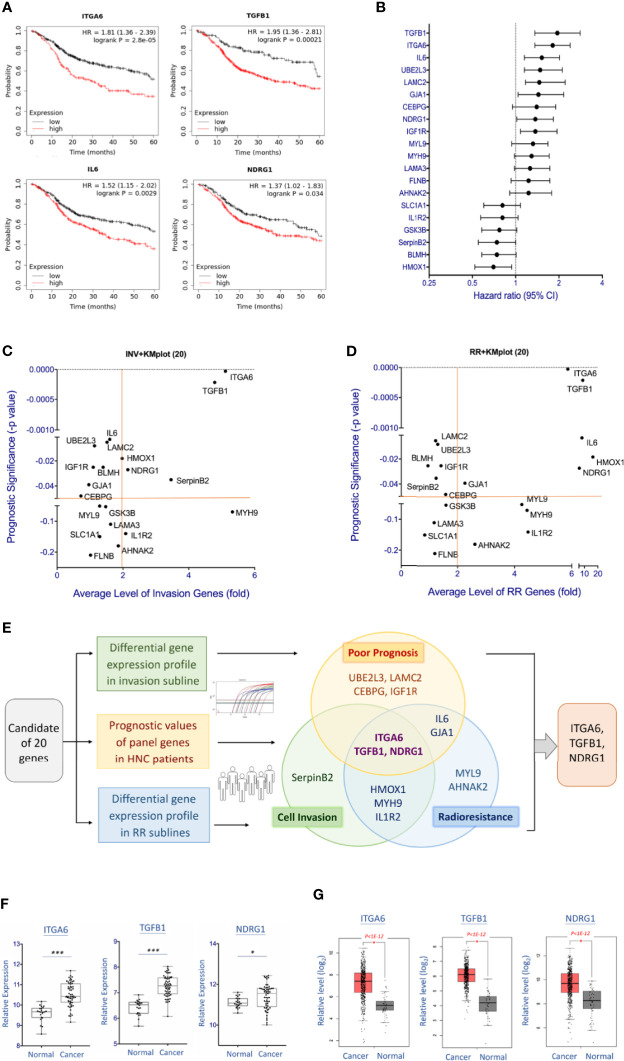
Molecular interplays between cell invasion and radioresistance that led to poor prognosis in HNC patients. **(A)** Prognostic significance of represent genes in HNC patients, as determined by Kaplan-Meier Plotter online tool using the head-neck squamous cell carcinoma dataset (n = 500). **(B)** The overall view of the prognostic vales of 20 genes as shown by the Hazard ratio with 95% confidence interval (CI), as determined by Kaplan-Meier Plotter using head-neck squamous cell carcinoma dataset (n = 500). **(C)** The overall view of 20 gene over-expressions (x-axis) in the invasion sublines and the prognostic significance (y-axis) in HNC patients of each gene. Value further to the right are signified higher levels of over-expression, and those toward to the top represent more significance with poor prognosis. **(D)** The overall view of 20 gene over-expressions (x-axis) in the radioresistant sublines and the prognostic significance (y-axis) in HNC patients of each gene. Value further to the right are signified higher levels of over-expression, and those toward to the top represent more significance with poor prognosis. **(E)** The diagram showing the overall and overlap genes that were over-expressed in the invasion subline, in the radioresistant subline, and associated with poor prognosis in HNC patients. Note that three molecules, ITGA6, TGFB1, and NDRG1, were recruited in these three parameters. **(F, G)** Relative levels of the gene expressions between oral mucosa specimens from healthy individuals (Normal) and oral cancer tissues from HNC patients (Cancer). The gene expression data, including ITGA6, TGFB1, and NDRG1 was retrieved from GEO Dataset GSE25099 **(F)** and TCGA-HNSC dataset **(G)**. (***p<0.001, *p<0.05, t-test).

From the insight of the clinical findings that cancer patients with metastasis or therapeutic resistance often led to poor prognosis, we examined the potential significance of the 20 co-regulatory molecules on HNC patients’ prognostic effects. We applied the KM-Plotter suit to analyze the association of gene expression levels and patients’ survival using the TCGA-HNSC cohort (n=500) (18). The patient characteristics of this cohort were summarized in **Supplementary Table S4**. **Figure 4A** showed few examples of the highly significant results. For each gene, the hazard ratio (HR) and P-value of the prognostic association were summarized in **Supplementary Table S2** and **Figure 4B**. As shown, many molecules exhibited good prediction power to worse prognosis, including ITGA6 (P=2.8E-05), ITGB1 (P=2.1E-04), IL6 (P=0.0029), and LAMC2 (P=0.0031), UBEL3 (P=0.0042), and NDRG1 (P=0.034).

To determine the association of clinical prognosis and gene expressions in the phenotypes of cell invasion or radioresistance, **Figures 4C, D** were plotted to show the associations of each gene. Although various clinically relevant genes were found between these two panels, several common molecules were found. **Figure 4E** summarized the molecules that were 2-fold over-expressed in the invasion or radioresistant cells and related to poor prognosis in HNC patients (P<0.05, HR>1.0). As shown, three molecules were distinguished out, as ITGA6, TGFB1, and NDRG1. These results represented a signature of functionally interplayed molecules between cell invasion and radioresistance and led to poor prognosis in HNC patients.

To further assess these three genes’ carcinogenic effect, we also examined the differential expression levels of these molecules between normal and tumor tissues using two microarray datasets, the GSE25099 and TCGA-HNSC (18, 22). The GSE25099 dataset contains a transcriptomic profile of 57 carcinoma tissues from oral cancer patients and 22 normal oral mucosa tissues from healthy individuals. The TCGA-HNSC dataset comprised 519 patients with head-neck squamous cell carcinoma and 44 normal tissues. **Figures 4F, G** showed the results. As shown, all these molecules were significantly over-expressed in the cancer patients in both assay cohorts. These results suggest that the molecules ITGA6, TGFB1, and NDRG1 contribute to cancer aggressiveness and participate in the malignant transformation from normal cells.

### ITGA6 Promoted Cell Invasion *via* Regulating the Integrity of Extracellular Matrix (ECM)

**Figure 5:**
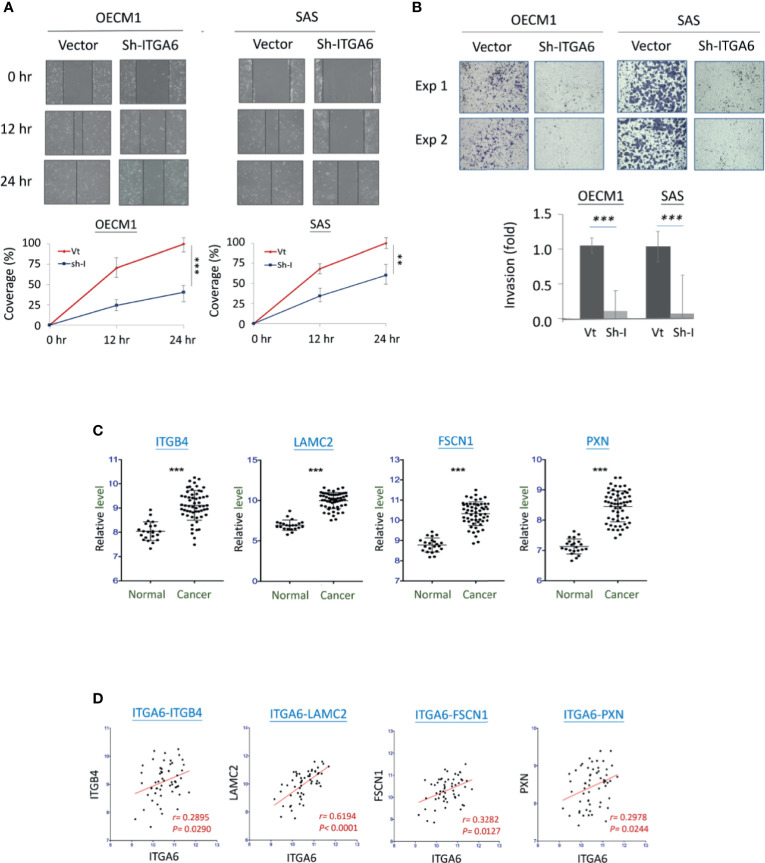
ITGA6 promoted cell motility *via* regulating the integrity of extracellular matrix (ECM). **(A)** ITGA6 silencing decreased cell migration. After transfection of ITGA6-shRNA plasmids, the HNC cells (OECM1, SAS) were subjected to *in vitro* wound healing assay. Cell migration toward the gap was observed, photographed, and quantified at the indicated times. **(B)** ITGA6 silencing attenuated cell invasion. After transfection of ITGA6-shRNA plasmids, the HNC cells (OECM1, SAS) were subjected to Matrigel invasion assay. The cells that invaded through the Matrigel-coated membranes to the reverse side were stained, photographed, and quantified. **(C)** Significant increases of ECM-associated gene expressions in the oral cancer tissues from HNC patients (Cancer) compared to the oral mucosa specimens from healthy individuals (Normal). The gene expression data, including ITGB4, LAMC2, FSCN1, and PXN, was retrieved from GEO Datasets GSE25099. **(D)** Correlative expressions between ITGA6 and ECM associated molecules ITGB4, LAMC2, FSCN1, and PXN, in the oral cancer tissues from HNC patients. The gene expression data was retrieved from GEO Datasets GSE25099. (**p < 0.01, ***P < 0.001, t-test).

ITGA6 showed at the authoritative place of cellular molecules in the aggressive phenotypes and worse clinical presentations; this molecule was selected for further mechanistic investigation. ITGA6 (Integrin alpha-6) is a heterodimeric component of the integrin receptor protein in epithelial cells, plays a critical role in maintaining the mechanical integrity of cell membrane for tissue architecture (23, 24). The cellular functions of ITGA6 related to cell invasion and radiosensitivity were assessed using shRNA stably knockdown experiments. The effects of cell migration and invasion were evaluated by *in vitro* wound healing and Matrigel invasion assays. As shown in **Figure 5A**, silencing ITGA6 resulted in a slower migration toward the gap area in two HNC cell lines by decreasing approximately to 40% and 60% in OECM1 and SAS cells at 24 hr. More apparently, ITGA6-silencing reduced cell invasion, with down to 10% and 8% in OECM1 and SAS cells (**Figure 5B**). These results suggested that ITGA6 functioned in promoting cell migration and invasion; silencing this molecule may inhibit cancer metastasis.

To examine whether ITGA6 function may relate to molecular presentation in clinical cancers, we further examined the association of ITGA6 expression level and motility-related molecules using an HNC microarray dataset GSE25099 (22). Several extracellular matrix (ECM) molecules were determined, including ITGB4, LAMC2, FSCN1, and PXN. The expression levels of these molecules were shown in **Figure 5C**. As shown, all these genes were significantly over-expressed in the cancer tissues compared to the normal tissues from healthy individuals (P<0.001 in all molecules). Furthermore, in the cancer tissues, these genes were all statistically correlated with the expression of ITGA6 (**Figure 5D**). These results suggest that ITGA6 promoted cell invasion *via* regulation of ECM integrity in HNC.

### ITGA6 Facilitated Radioresistance Through Regulation of the Apoptotic Related Mechanism

**Figure 6:**
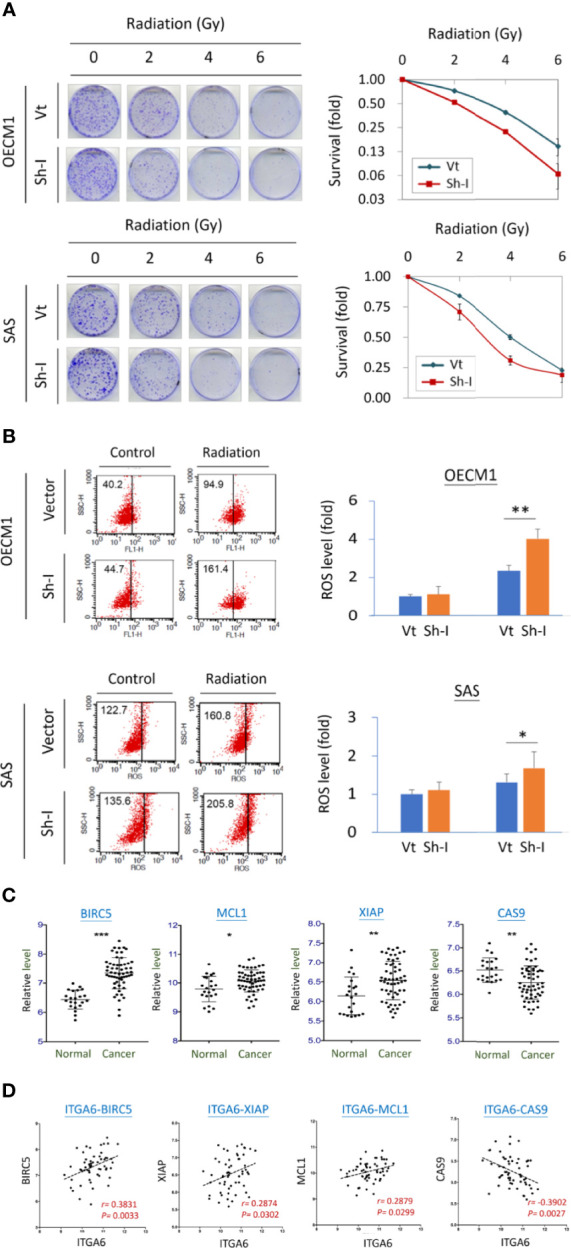
ITGA6 facilitated radioresistance through regulation of the apoptotic related mechanism. **(A)** ITGA6 silencing increased radiosensitivity. After transfection of ITGA6-shRNA plasmids, the HNC cells (OECM1, SAS) were subjected to clonogenic survival assay. The colony survival fractions were determined after the cells were irradiated with various doses (0 to 6 Gy). **(B)** ITGA6 silencing increased ROS production in HNC cells. After transfection ITGA6-shRNA plasmids, the HNC cells (OECM1, SAS) were subjected to irradiation. The ROS level was determined using H2DCF-DA oxidation method and analyzed by flow cytometry. **(C)** Significantly higher expressions of survival related genes (BIRC5, MCL1, XIAP) and lower expression of apoptotic gene (CAS9) in the oral cancer tissues from HNC patients (Cancer) compared to the oral mucosa specimens from healthy individuals (Normal). The gene expression data was retrieved from GEO Datasets GSE25099. (***p < 0.001, **p < 0.01, *p < 0.05, t-test). **(D)** Correlative expressions between ITGA6 and survival or apoptotic associated molecules BIRC5, MCL1, XIAP, and CAS9, in the oral cancer tissues from HNC patients. The gene expression data was retrieved from GEO Datasets GSE25099.

The potential effect of ITGA6 on radiosensitivity was determined by clonogenic survival assay. As shown in **Figure 6A**, ITGA6-silencing reduced radioresistance by decreasing colony survival to 42% and 61%, respectively, at 6 Gy in OECM1 and 4 Gy in SAS cells. It is well established that ionizing radiation can induce ROS in the cell resulting in apoptosis (20). We further determined whether ITGA6 contributing to radioresistance may relate to ROS regulation. The intracellular ROS was measured using the H2DCF-DA oxidation method, and the green fluorescence DCF product was analyzed by flow cytometry (20). Results were shown in **Figure 6B**. Without irradiation, ITGA6-silencing had minimal effect on intracellular ROS level. Irradiation significantly induced ROS production in either vector- or sh-ITGA6 transfected cells. However, the ITGA6-silencing cells increased more considerably than the controls, by 1.7- and 1.3-fold higher in the OECM1 and SAS cell lines. These results suggested that ITGA6 contributed to radioresistance *via* inhibition of the ROS generation pathway. Silencing ITGA6 may reverse radioresistance by sensitizing cancer cells to radiotherapy.

We also assessed the potential association of ITGA6 with the clinical presentation of survival-related molecules using a microarray dataset GSE25099 (22). These molecules included BIRC5, MCL1, XIAP, and apoptotic gene CAS9. The expression levels of these molecules were shown in **Figure 6C**. As shown, these survival genes (BIRC5, MCL1, XIAP) were significantly increased expressions in the cancer tissues, while the apoptotic gene (CAS9) was reduced compared to the normal tissues from healthy individuals. Furthermore, in the cancer tissues, these genes were all statistically correlated with the expression of ITGA6 (**Figure 6D**). These results suggest that ITGA6 facilitated radioresistance by reducing cellular ROS level leading to anti-apoptotic or survival advantage in HNC.

## Discussion

**Figure 7:**
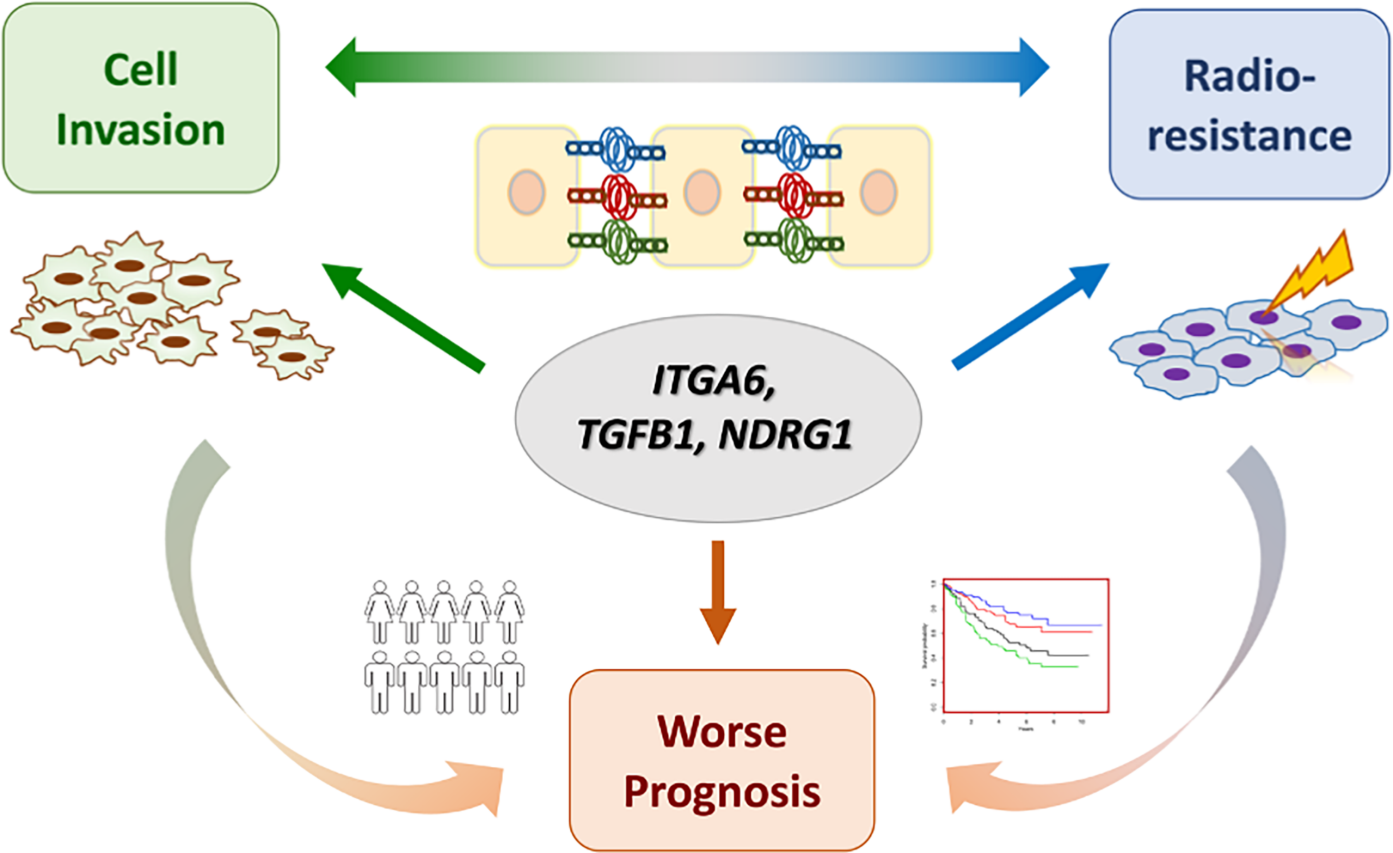
A model of molecular crosstalk between cell invasion and radioresistance in HNC.

Cancer metastasis and recurrence after radiotherapy are the major causes of treatment failure in HNC. It is commonly found that patients with either condition may accompany the outcome of the other. In this study, we revealed the molecular interplays of cell invasion and radioresistance in HNC, aiming to discover effective biomarkers for the intervention of aggressive cancer at one time. Our works presented in this study can be highlighted by few points (**Figure 7**). (1) The phenotypic crosstalk between cell invasion and radioresistance was confirmed in HNC cells. (2) The functional pathways co-regulate between these two phenotypes were established. The focal adhesion was revealed to play a significant role in contributing to these attributes. (3) The molecular interplays between cell invasion and radioresistance were identified, as ITGA6, TGFB1, and NDRG1, further leading to poor prognosis in HNC. (4) ITGA6 was demonstrated to play an imperative role in these aggressive cancer phenotypes. It may occur through modulation of ECM or anti-apoptotic mechanism to achieve cell invasion and radioresistance. Silencing this molecule suppressed cell migration, invasion, and attenuated radioresistance; this molecule may be used as a molecular target for refractory HNC treatment. In the future, further validation studies with protein expression levels in clinical subjects are highly recommended to confirm these results.

In this study, several functional pathways were identified cross-regulating cell invasion and radioresistance in HNC. Interesting, the functional mechanisms related to motility comprised the most, as focal adhesion, proteoglycans in cancer, ECM-receptor interaction, and actin cytoskeleton regulation (**Figure 2B**). Although these motility-related mechanisms were well accepted to regulate cell invasion, they were noted to modulate radioresistance in the present study. The integrity of ECM and focal adhesion mechanism were important in response to radiation stress for cellular survival. Three signaling pathways were identified for the network molecules to critically co-regulate these two phenotypes, PI3-AKT, Rap1, and MAPK (**Figure 2B**). PI3K-Akt is an intracellular signaling pathway that mediator of several membrane-bound receptor tyrosine kinases (25). In response to extracellular stimuli, its activation may induce downstream oncogenic pathways to promote cancer aggressiveness. Consistent with our findings, this pathway has been reported to participate in cell invasion, cell proliferation, and therapeutic resistance (26, 27). The MAPK family proteins include three major signaling molecules, ERK, p38 kinase, and JNK, that transduce extracellular signaling into nuclei following turn-on gene expression (28). Since MAPK may induce multiple downstream signals, it regulates a wide range of cellular functions, including cell proliferation, differentiation, apoptosis, and stress response (28, 29). Our finding of MAPK signaling pathway in co-regulation of two aggressive cancer phenotypes agreed with these reports. The Rap1 protein is a small GTPase protein belonging to the RAS oncogene family. It acts as molecular switches between an inactive GDP-bound and an active GTP-bound conformation to turn on signal transduction (30). Rap1 is important for molecular junction and cell adhesion, which is significantly associated with cell invasion and cancer metastasis (30, 31). Furthermore, the Ras family has also been reported regulating cell proliferation and survival (30, 31). This growth supportive function may explain the radioresistant mechanism of the Rap1 pathway noted in this study. Thus, activation of these pathways may contribute to a more aggressive cancer, which is correlated with our findings related to cell invasion and radioresistant phenotypes of HNC.

In searching panel molecules co-regulating cell invasion and radioresistance and contributing to poor prognosis in HNC, three molecules were prominent, as ITGA6, TGFB1, and NDRG1 (**Figure 4E**). NDRG1 is a multifunctional protein that participates in several cellular processes, including cellular differentiation, stress response, and apoptosis (32, 33). Reports of NDRG1 in modulating tumor development are inconsistent. NDRG1 may act as an oncogene, for it has been reported to be overexpressed in many types of cancers, including bladder, liver, lung, and colorectal cancers (34–37). The oncogenic function of NDRG1 includes promoting cellular motility, tumorigenesis, and therapeutic resistance (36–41). Paradoxically, NDRG1 is also a putative tumor suppressor since it has been found downregulated in several types of cancers, such as prostate, pancreatic, and endometrial cancers (42–44). The reported tumor-suppressive functions were on the suppression of cell growth and motility (43–45). The opposite effects of NDRG1 in modulating malignancy may depend on the cells under certain conditions or the specific types of tissues. In the present study, we found that NDRG1 was upregulated in the invasive and radioresistant sublines (**Figure 3**), over-expressed in the cancer tissues (**Figure 4F**), and associated with poor prognosis in HNC patients (**Figure 4A**). Our results, in agreement with the oncogenic reports, suggested that this molecule modulates multiple malignant functions in HNC.

TGFB1 is a polypeptide member of the transforming growth factor-beta (TGFB) superfamily, a cytokine that predominantly exists in the tumor microenvironment (46). This molecule is mostly considered an oncogene because it was overexpressed in several cancers and associated with a poor prognosis (47–49). Mechanically, TGFB1 was presumably modulating malignant function *via* suppression of immunosurveillance (50). Recently, this molecule has been reported contributing to malignancy *via* induction of cellular motility through multiple mechanisms. These include the activation of epithelial-mesenchymal transition (51), modulating focal adhesion structure *via* interacting with laminin family molecules (52) or interacting with tyrosine kinase receptor to induce oncogenic signaling (53). Consistently with these reports, we found that TGFB1 was markedly upregulated in the invasive sublines (**Figure 3**), overexpressed in the cancer tissues (**Figure 4F**), and associated with poor prognosis in HNC patients (**Figure 4A**). We further noted that TGFB1 facilitated radioresistant in HNC cells, and which was not previously reported in our knowledge (**Figure 3**). Thus, our results supported previous findings and provided a novel functional mechanism of TGFB1 in cancer aggressiveness. Science ITGB1 is a secretory protein; this molecule may be used as a circulating tumor marker for prognostic application.

ITGA6 (Integrin alpha-6), also named VLA-6 and CD49f, encodes a member of the integrin alpha-6 subunit protein (23, 24). Integrins are heterodimeric receptors that comprise paired α and β subunits. There are 18 α and 8 β subunits in the human genome that combine to provide 24 integrin receptors, each with its specificity for selected extracellular matrix (24). Integrin α6β4 is a cellular adhesion molecule that binds to its ligand laminins in epithelial cells and plays a critical structural role in the hemidesmosome (24, 54). Although integrin’s primary function is to maintain cell membranes’ mechanical integrity to maintain tissue architecture, recent studies have shown many more biological roles than what was initially thought. Through interaction with ligand laminin or cell-surface receptor protein, integrin may induce several downstream signal pathways, including FAK, EGFR, and AKT oncogenic mechanisms (54–56). Clinically, ITGA6 has been reported altered expression in several cancers. Over-expression of this molecule was found in several types of cancers, and this up-regulation was associated with poor prognosis (56, 57). Mechanistically, this molecule has been reported to participate in several malignant functions, including cell proliferation, cell motility, and drug resistance (58–60). All these reports were consistent with our findings in HNC. We showed that a high level of ITGA6 was overexpressed in cancer tissues (**Figure 4F**) and associated with a worse prognosis in HNC patients (**Figure 4A**). Although ITGA6 was expected to promote cell invasion, we also revealed its function in facilitating radioresistance (**Figure 6A**). This result was supported by the previous finding in breast cancer that ITGA6 plays a critical role in radioresistance *via* regulating Akt/Erk signaling pathway (61). We also showed that this function could be achieved *via* modulation of intracellular ROS levels (**Figure 6B**) and leading to anti-apoptotic advantage (**Figure 6D**). At the molecular level, we demonstrated that ITGA6 facilitated radioresistance *via* regulating the apoptotic-related mechanism. This finding was shown by the correlative expressions of ITGA6 with a panel of survival genes, including BIRC5, MCL1, XIAP (**Figure 6D**). Note that BIRC5 has been reported regulating radioresistance and metastasis (62, 63), thus further supporting our finding of this molecule on the cross regulatory function in these two phenotypes. Silencing this molecule reversed malignant presentation significantly, as attenuation of invasion ability (**Figure 5**) and induction of radio-sensitization in HNC (**Figure 6**). Thus, ITGA6 may serve as a predictive marker of radioresistance, a prognostic marker of metastasis, and a molecular target for developing a therapeutic modality for the treatment of refractory cancers.

In conclusion, the poor prognosis of HNC patients was often resulted from cancer metastasis or therapeutic resistance. In this study, we have employed a systemic approach by elucidation of the molecular interplays between cell invasion and radioresistance, aiming to identify prominent molecules contributing to the prognosis of HNC. We revealed phenotypic crosstalk between cell invasion and radioresistance, determined the functional pathways (such as focal adhesion) co-regulating these two phenotypes, and identified a panel of interplay molecules leading to poor prognosis (ITGA6, TGFB1, and NDRG1). A hub molecule ITGA6 was demonstrated to play an imperative role contributing to the aggressive phenotypes. Silencing this molecule suppressed cell migration, invasion, and attenuated radioresistance. These panel molecules, such as ITGA6, may serve as prognostic markers of metastasis, predictive markers of radioresistance, and molecular therapeutic targets to treat refractory HNC.

## Data Availability Statement

The original contributions presented in the study are included in the article/**Supplementary Material**. Further inquiries can be directed to the corresponding author.

## Author Contributions

Conceptualization, G-RY, JC, and A-JC. Methodology, G-RY, Y-LL, Y-JC, and A-JC. Software, G-RY, Y-LL, and Y-JC. Validation, G-RY, Y-LL, Y-JC, and A-JC. Formal analysis, G-RY, Y-LL, Y-JC, and A-JC. investigation G-RY, Y-LL, Y-JC, and A-JC. Resources, G-RY, JC, Y-CH, K-HF, Y-CC, C-JK, and A-JC. Data curation, G-RY, JC, Y-LL, Y-JC, and A-JC. Writing—original draft preparation, G-RY, JC, and A-JC. Writing—review and editing, G-RY, JC, Y-LL, Y-JC, Y-CH, K-HF, Y-CC, C-JK, and A-JC. Visualization, G-RY, JC, and A-JC. Supervision, JC,Y-CH, K-HF, Y-CC, C-JK, and A-JC. Project administration, JC and A-JC. Funding acquisition, JC and A-JC. All authors contributed to the article and approved the submitted version.

## Funding

This research was supported by Ministry of Science and Technology (Most-108-2320-B-182-029-MY3), and Chang Gung Memorial Hospital- Linko Medical Center (CMRPD1H0481~3, XPRPG3H0131~3).

## Conflict of Interest

The authors declare that the research was conducted in the absence of any commercial or financial relationships that could be construed as a potential conflict of interest.

## References

[1] Chow LQM. Head and Neck Cancer. N Engl J Med (2020) 382:60–72. 10.1056/NEJMra1715715 31893516

[2] Huang SH O’Sullivan B. Overview of the 8th Edition TNM Classification for Head and Neck Cancer. Curr Treat Options Oncol (2017) 18:40. 10.1007/s11864-017-0484-y 28555375

[3] Li YC Cheng AJ Lee LY Huang YC Chang JT. Multifaceted Mechanisms of Areca Nuts in Oral Carcinogenesis: The Molecular Pathology From Precancerous Condition to Malignant Transformation. J Cancer (2019) 10:4054–62. 10.7150/jca.29765 PMC6692602 31417650

[4] Chang WC Lin CS Yang CY Lin CK Chen YW. Lymph Node Density as a Prognostic Predictor in Patients With Betel Nut-Related Oral Squamous Cell Carcinoma. Clin Oral Investig (2018) 22:1513–21. 10.1007/s00784-017-2247-3 PMC5866838 29038963

[5] Arosio AD Pignataro L Gaini RM Garavello W. Neck Lymph Node Metastases From Unknown Primary. Cancer Treat Rev (2017) 53:1–9. 10.1016/j.ctrv.2016.11.014 28027480

[6] Ginos MA Page GP Michalowicz BS Patel KJ Volker SE Pambuccian SE. Identification of a Gene Expression Signature Associated With Recurrent Disease in Squamous Cell Carcinoma of the Head and Neck. Cancer Res (2004) 64:55–63. 10.1158/0008-5472.can-03-2144 14729608

[7] Erdem NF Carlson ER Gerard DA. Characterization of Gene Expression Profiles of 3 Different Human Oral Squamous Cell Carcinoma Cell Lines With Different Invasion and Metastatic Capacities. J Oral Maxillofac Surg (2008) 66:918–27. 10.1016/j.joms.2007.12.036 18423281

[8] Kang CJ Chen YJ Liao CT Wang HM Chang JT Lin CY. Transcriptome Profiling and Network Pathway Analysis of Genes Associated With Invasive Phenotype in Oral Cancer. Cancer Lett (2009) 284:131–40. 10.1016/j.canlet.2009.04.014 19457608

[9] Chiu CC Lin CY Lee LY Chen YJ Lu YC Wang HM. Molecular Chaperones as a Common Set of Proteins That Regulate the Invasion Phenotype of Head and Neck Cancer. Clin Cancer Res (2011) 17:4629–41. 10.1158/1078-0432.CCR-10-2107 21642380

[10] Ogawa K Utsunomiya T Mimori K Tanaka F Haraguchi N Inoue H. Differential Gene Expression Profiles of Radioresistant Pancreatic Cancer Cell Lines Established by Fractionated Irradiation. Int J Oncol (2006) 28:705–13. 10.3892/ijo.28.3.705 16465376

[11] Fukuda K Sakakura C Miyagawa K Kuriu Y Kin S Nakase Y. Differential Gene Expression Profiles of Radioresistant Oesophageal Cancer Cell Lines Established by Continuous Fractionated Irradiation. Br J Cancer (2004) 91:1543–50. 10.1038/sj.bjc.6602187 PMC2409931 15365572

[12] Lin TY Chang JT Wang HM Chan SH Chiu CC Lin CY. Proteomics of the Radioresistant Phenotype in Head-And-Neck Cancer: Gp96 as a Novel Prediction Marker and Sensitizing Target for Radiotherapy. Int J Radiat Oncol Biol Phys (2010) 78:246–56. 10.1016/j.ijrobp.2010.03.002 20615631

[13] You GR Cheng AJ Lee LY Huang YC Liu H Chen YJ. Prognostic Signature Associated With Radioresistance in Head and Neck Cancer *Via* Transcriptomic and Bioinformatic Analyses. BMC Cancer (2019) 19:64. 10.1186/s12885-018-5243-3 30642292 PMC6332600

[14] Lorini L Ardighieri L Bozzola A Romani C Bignotti E Buglione M. Prognosis and Management of Recurrent and/or Metastatic Head and Neck Adenoid Cystic Carcinoma. Oral Oncol (2021) 115:105213. 10.1016/j.oraloncology.2021.105213 33578204

[15] Liu JK Liu HF Ding Y Gao GD. Predictive Value of microRNA Let-7a Expression for Efficacy and Prognosis of Radiotherapy in Patients With Lung Cancer Brain Metastasis: A Case-Control Study. Med (Baltimore) (2018) 97:e12847. 10.1097/MD.0000000000012847 PMC6221706 30383637

[16] Tang E Nguyen TV Clatot F Rambeau A Johnson A Sun XS. Radiation Therapy on Primary Tumour of Synchronous Metastatic Head and Neck Squamous Cell Carcinomas. Cancer Radiother (2020) 24:559–66. 10.1016/j.canrad.2020.05.004 32753240

[17] Higgins GS O’Cathail SM Muschel RJ McKenna WG. Drug Radiotherapy Combinations: Review of Previous Failures and Reasons for Future Optimism. Cancer Treat Rev (2015) 41:105–13. 10.1016/j.ctrv.2014.12.012 25579753

[18] Nagy A Munkárcsy G Győrffy B. Pancancer Survival Analysis of Cancer Hallmark Genes. Sci Rep (2021) 11:6047. 10.1038/s41598-021-84787-5 33723286 PMC7961001

[19] Li YC Chang JT Chiu C Lu YC Li YL Chiang CH. Areca Nut Contributes to Oral Malignancy Through Facilitating the Conversion of Cancer Stem Cells. Mol Carcinog (2016) 55:1012–23. 10.1002/mc.22344 26087469

[20] Li YL Chang JT Lee LY Fan KH Lu YC Li YC. GDF15 Contributes to Radioresistance and Cancer Stemness of Head and Neck Cancer by Regulating Cellular Reactive Oxygen Species *Via* a SMAD-Associated Signaling Pathway. Oncotarget (2017) 8:1508–28. 10.18632/oncotarget.13649 PMC5352073 27903972

[21] Pawlik TM Keyomarsi K. Role of Cell Cycle in Mediating Sensitivity to Radiotherapy. Int J Radiat Oncol Biol Phys (2004) 59:928–42. 10.1016/j.ijrobp.2004.03.005 15234026

[22] Peng CH Liao CT Peng SC Chen YJ Cheng AJ Juang JL. A Novel Molecular Signature Identified by Systems Genetics Approach Predicts Prognosis in Oral Squamous Cell Carcinoma. PloS One (2011) 6:e23452. 10.1371/journal.pone.0023452 21853135 PMC3154947

[23] Hynes RO. Integrins: Bidirectional, Allosteric Signaling Machines. Cell (2002) 110:673–87. 10.1016/s0092-8674(02)00971-6 12297042

[24] Das V Kalyan G Hazra S Pal M. Understanding the Role of Structural Integrity and Differential Expression of Integrin Profiling to Identify Potential Therapeutic Targets in Breast Cancer. J Cell Physiol (2018) 233:168–85. 10.1002/jcp.25821 28120356

[25] Martini M De Santis MC Braccini L Gulluni F Hirsch E. PI3K/AKT Signaling Pathway and Cancer: An Updated Review. Ann Med (2014) 46:372–83. 10.3109/07853890.2014.912836 24897931

[26] Toulany M Rodemann HP. Phosphatidylinositol 3-Kinase/Akt Signaling as a Key Mediator of Tumor Cell Responsiveness to Radiation. Semin Cancer Biol (2015) 35:180–90. 10.1016/j.semcancer.2015.07.003 26192967

[27] Perri F Pacelli R Della Vittoria Scarpati G Cella L Giuliano M Caponigro F. Radioresistance in Head and Neck Squamous Cell Carcinoma: Biological Bases and Therapeutic Implications. Head Neck (2015) 37:763–70. 10.1002/hed.23837 24995469

[28] Burotto M Chiou VL Lee JM Kohn EC. The MAPK Pathway Across Different Malignancies: A New Perspective. Cancer (2014) 120:3446–56. 10.1002/cncr.28864 PMC4221543 24948110

[29] Liu F Yang X Geng M Huang M. Targeting ERK, an Achilles’ Heel of the MAPK Pathway, in Cancer Therapy. Acta Pharm Sin B (2018) 8:552–62. 10.1016/j.apsb.2018.01.008 PMC6089851 30109180

[30] Jaskiewicz A Pajak B Orzechowski A. The Many Faces of Rap1 Gtpase. Int J Mol Sci (2018) 19:2848. 10.3390/ijms19102848 PMC6212855 30241315

[31] Shah S Brock EJ Ji K Mattingly RR. Ras and Rap1: A Tale of Two Gtpases. Semin Cancer Biol (2019) 54:29–39. 10.1016/j.semcancer.2018.03.005 29621614 PMC6170734

[32] Kitowska A Pawelczyk T. N-Myc Downstream Regulated 1 Gene and its Place in the Cellular Machinery. Acta Biochim Pol (2010) 57:15–21. 10.18388/abp.2010_2367 20300662

[33] Song Y Cao L. N-Myc Downstream-Regulated Gene 1: Diverse and Complicated Functions in Human Hepatocellular Carcinoma (Review). Oncol Lett (2013) 6:1539–42. 10.3892/ol.2013.1636 PMC3834550 24260043

[34] Li A Zhu X Wang C Yang S Qiao Y Qiao R. Upregulation of NDRG1 Predicts Poor Outcome and Facilitates Disease Progression by Influencing the EMT Process in Bladder Cancer. Sci Rep (2019) 9:5166. 10.1038/s41598-019-41660-w 30914736 PMC6435802

[35] Dai T Dai Y Murata Y Husni RE Nakano N Sakashita S. The Prognostic Significance of N-Myc Downregulated Gene 1 in Lung Adenocarcinoma. Pathol Int (2018) 68:224–31. 10.1111/pin.12644 29431240

[36] Song Y Lv L Du J Yue L Cao L. Correlation of N-Myc Downstream-Regulated Gene 1 Subcellular Localization and Lymph Node Metastases of Colorectal Neoplasms. Biochem Biophys Res Commun (2013) 439:241–6. 10.1016/j.bbrc.2013.08.049 23973486

[37] Liu Y Wang D Li Y Yan S Dang H Yue H. Long Noncoding RNA CCAT2 Promotes Hepatocellular Carcinoma Proliferation and Metastasis Through Up-Regulation of NDRG1. Exp Cell Res (2019) 379:19–29. 10.1016/j.yexcr.2019.03.029 30922920

[38] Chang X Xu X Ma J Xue X Li Z Deng P. NDRG1 Expression Is Related to the Progression and Prognosis of Gastric Cancer Patients Through Modulating Proliferation, Invasion and Cell Cycle of Gastric Cancer Cells. Mol Biol Rep (2014) 41:6215–23. 10.1007/s11033-014-3501-2 24985974

[39] Wang Y Zhou Y Tao F Chai S Xu X Yang Y. N-Myc Downstream Regulated Gene 1(NDRG1) Promotes the Stem-Like Properties of Lung Cancer Cells Through Stabilized C-Myc. Cancer Lett (2017) 401:53–62. 10.1016/j.canlet.2017.04.031 28456659

[40] Kim SC Shin YK Kim YA Jang SG Ku JL. Identification of Genes Inducing Resistance to Ionizing Radiation in Human Rectal Cancer Cell Lines: Re-Sensitization of Radio-Resistant Rectal Cancer Cells Through Down Regulating Ndrg1. BMC Cancer (2018) 18:594. 10.1186/s12885-018-4514-3 29801473 PMC5970486

[41] Zhang D Jia J Zhao G Yue M Yang H Wang J. NDRG1 Promotes the Multidrug Resistance of Neuroblastoma Cells With Upregulated Expression of Drug Resistant Proteins. BioMed Pharmacother (2015) 76:46–51. 10.1016/j.biopha.2015.10.015 26653549

[42] Lv XH Chen JW Zhao G Feng ZZ Yang DH Sun WW. N-Myc Downstream-Regulated Gene 1/Cap43 may Function as Tumor Suppressor in Endometrial Cancer. J Cancer Res Clin Oncol (2012) 138:1703–15. 10.1007/s00432-012-1249-4 PMC11824235 22678098

[43] Angst E Dawson DW Stroka D Gloor B Park J Candinas D. N-Myc Downstream Regulated Gene-1 Expression Correlates With Reduced Pancreatic Cancer Growth and Increased Apoptosis *In Vitro* and *In Vivo* . Surgery (2011) 149:614–24. 10.1016/j.surg.2010.11.002 21236457

[44] Li Y Pan P Qiao P Liu R. Downregulation of N-Myc Downstream Regulated Gene 1 Caused by the Methylation of CpG Islands of NDRG1 Promoter Promotes Proliferation and Invasion of Prostate Cancer Cells. Int J Oncol (2015) 47:1001–8. 10.3892/ijo.2015.3086 26202882

[45] Cen G Zhang K Cao J Qiu Z. Downregulation of the N-Myc Downstream Regulated Gene 1 Is Related to Enhanced Proliferation, Invasion and Migration of Pancreatic Cancer. Oncol Rep (2017) 37:1189–95. 10.3892/or.2017.5355 28075464

[46] Zarzynska JM. Two Faces of TGF-Beta1 in Breast Cancer. Mediators Inflamm (2014) 2014:141747. 10.1155/2014/141747 24891760 PMC4033515

[47] Zhang X Zhang P Shao M Zang X Zhang J Mao F. SALL4 Activates TGF-Beta/SMAD Signaling Pathway to Induce EMT and Promote Gastric Cancer Metastasis. Cancer Manag Res (2018) 10:4459–70. 10.2147/CMAR.S177373 PMC6188178 30349378

[48] Nishimura T Tamaoki M Komatsuzaki R Oue N Taniguchi H Komatsu M. SIX1 Maintains Tumor Basal Cells *Via* Transforming Growth Factor-Beta Pathway and Associates With Poor Prognosis in Esophageal Cancer. Cancer Sci (2017) 108:216–25. 10.1111/cas.13135 PMC5329162 27987372

[49] Karlicic V Vukovic J Stanojevic I Sotirovic J Peric A Jovic M. Association of Locally Produced IL10 and TGFb1 With Tumor Size, Histological Type and Presence of Metastases in Patients With Lung Carcinoma. J BUON (2016) 21:1210–8. 27837625

[50] Liu M Kuo F Capistrano KJ Kang D Nixon BG Shi W. TGF-Beta Suppresses Type 2 Immunity to Cancer. Nature (2020) 587:115–20. 10.1038/s41586-020-2836-1 PMC8347705 33087928

[51] Lee SJ Yang CS Kim DD Kang YN Kwak SG Park JB. Microenvironmental Interactions and Expression of Molecular Markers Associated With Epithelial-To-Mesenchymal Transition in Colorectal Carcinoma. Int J Clin Exp Pathol (2015) 8:14270–82. PMC4713529 26823743

[52] Chakrabarty S Liu BR Rajagopal S. Disruption of Transforming Growth Factor Beta-Regulated Laminin Receptor Function by Expression of Antisense Laminin, a Chain RNA in Human Colon Cancer Cells. J Cell Physiol (2001) 186:47–52. 10.1002/1097-4652(200101)186:1<47::AID-JCP1009>3.0.CO;2-A 11147813

[53] Shi Q Chen YG. Interplay Between TGF-Beta Signaling and Receptor Tyrosine Kinases in Tumor Development. Sci China Life Sci (2017) 60:1133–41. 10.1007/s11427-017-9-173-5 29067649

[54] Desgrosellier JS Cheresh DA. Integrins in Cancer: Biological Implications and Therapeutic Opportunities. Nat Rev Cancer (2010) 10:9–22. 10.1038/nrc2748 20029421 PMC4383089

[55] Pandolfi F Franza L Altamura S Mandolini C Cianci R Ansari A. Integrins: Integrating the Biology and Therapy of Cell-Cell Interactions. Clin Ther (2017) 39:2420–36. 10.1016/j.clinthera.2017.11.002 29203050

[56] Bianconi D Unseld M Prager GW. Integrins in the Spotlight of Cancer. Int J Mol Sci (2016) 17:2037. 10.3390/ijms17122037 PMC5187837 27929432

[57] Stewart RL O’Connor KL. Clinical Significance of the Integrin Alpha6beta4 in Human Malignancies. Lab Invest (2015) 95:976–86. 10.1038/labinvest.2015.82 PMC4554527 26121317

[58] Cooper J Giancotti FG. Integrin Signaling in Cancer: Mechanotransduction, Stemness, Epithelial Plasticity, and Therapeutic Resistance. Cancer Cell (2019) 35:347–67. 10.1016/j.ccell.2019.01.007 PMC6684107 30889378

[59] Phillips JL Taberlay PC Woodworth AM Hardy K Brettingham-Moore KH Dickinson JL. Distinct Mechanisms of Regulation of the ITGA6 and ITGB4 Genes by RUNX1 in Myeloid Cells. J Cell Physiol (2018) 233:3439–53. 10.1002/jcp.26197 28926098

[60] Bigoni-Ordonez GD Czarnowski D Parsons T Madlambayan GJ Villa-Diaz LG. Integrin Alpha6 (CD49f), The Microenvironment and Cancer Stem Cells. Curr Stem Cell Res Ther (2019) 14:428–36. 10.2174/1574888X13666181002151330 30280675

[61] Hu T Zhou R Zhao Y Wu G. Inteegrin α6/Akt/Erk Signaling Is Essential for Human Breast Cancer Resistance to Radiotherapy. Sci Rep (2016) 6:33376. 10.1038/srep33376 27624978 PMC5022055

[62] Rödel F Reichert S Sprenger T Gaipl US Mirsch J Liersch T. The Role of Survivin for Radiation Oncology: Moving Beyoud Apoptosis Inhibition. Curr Med Chem (2011) 18:191–9. 10.2174/092986711794088362 21110807

[63] Mehrotra S Languino LR Raskett CM Mercurio AM Dohi T Altieri DC. IAP Regulation of Metastasis. Cancer Cell (2010) 17:53–64. 10.1016/j.ccr.2009.11.021 20129247 PMC2818597

