# Supplementary material for: Molecular Interplays Between Cell Invasion and Radioresistance That Lead to Poor Prognosis in Head-Neck Cancer
Source: Front Oncol. 2021 Jul 9;11:681717. doi: 10.3389/fonc.2021.681717 (PMC8299304; doi:10.3389/fonc.2021.681717)
Supplement: Supplementary file 1 [file Table_1.docx]

**Supplementary Table S1|** List of the differentially expressed genes associated with both cell invasion and radioresistant phenotypes in three HNC cell lines, as determined by microarray analysis. The average fold change and P-value were listed.

|  | Gene name | Invasion subline | | RR subline | |
| --- | --- | --- | --- | --- | --- |
| Gene symbol |  | Expression (Avg. fold) | P-value | Expression (Avg. fold) | P-value |
| AHNAK2 | AHNAK Nucleoprotein 2 | 1.5883 | <0.0001 | 4.2202 | <0.0001 |
| BLMH | Bleomycin Hydrolase | 1.7005 | <0.0001 | 1.8116 | 0.0213 |
| CEBPG | CCAAT Enhancer Binding Protein Gamma | 1.5934 | <0.0001 | 1.7902 | 0.0018 |
| GJA1 | Gap Junction Protein Alpha 1 | 2.2796 | 0.0059 | 4.6354 | 0.0373 |
| GSK3B | Glycogen Synthase Kinase 3 Beta | 1.6906 | <0.0001 | 1.6569 | 0.0195 |
| HMOX1 | Heme Oxygenase 1 | 1.6671 | <0.0001 | 1.9433 | 0.0269 |
| IGF1R | Insulin Like Growth Factor 1 Receptor | 1.5944 | 0.0029 | 1.6889 | 0.0026 |
| IL1R2 | Interleukin 1 Receptor Type 2 | 1.6349 | 0.0015 | 1.6674 | 0.0482 |
| LAMA3 | Laminin Subunit Alpha 3 | 1.7915 | 0.0001 | 4.9970 | 0.0238 |
| LAMC2 | Laminin Subunit Gamma 2 | 1.8326 | <0.0001 | 3.0170 | 0.0088 |
| MYH9 | Myosin Heavy Chain 9 | 1.5210 | <0.0001 | 1.7404 | 0.0054 |
| MYL9 | Myosin Light Chain 9 | 1.8417 | 0.0028 | 2.5140 | 0.0301 |
| NDRG1 | N-Myc Downstream Regulated 1 | 1.5269 | 0.0157 | 5.9977 | 0.0014 |
| SerpinB2 | Serpin Family B Member 2 | 1.8546 | 0.0001 | 2.3785 | 0.0071 |
| SLC1A1 | Solute Carrier Family 1 Member 1 | 1.9702 | 0.0008 | 1.8941 | 0.0071 |
| UBE2L3 | Ubiquitin Conjugating Enzyme E2 L3 | 1.5459 | <0.0001 | 1.5476 | 0.0008 |
| B3GALNT1 | Beta-1,3-N-Acetylgalactosaminyltransferase 1 | 0.6466 | 0.0001 | 0.6296 | 0.0493 |
| DLEU2 | Deleted In Lymphocytic Leukemia 2 | 0.4898 | <0.0001 | 0.6584 | 0.0049 |
| FNDC3A | Fibronectin Type III Domain Containing 3A | 0.6341 | <0.0001 | 0.6637 | <0.0001 |
| HMGN4 | High Mobility Group Nucleosomal Binding Domain 4 | 0.5512 | 0.0041 | 0.6225 | 0.0023 |
| SLC48A1 | Solute Carrier Family 48 Member 1 | 0.5757 | <0.0001 | 0.6440 | 0.0149 |
