# Supplementary material for: Molecular Interplays Between Cell Invasion and Radioresistance That Lead to Poor Prognosis in Head-Neck Cancer
Source: Front Oncol. 2021 Jul 9;11:681717. doi: 10.3389/fonc.2021.681717 (PMC8299304; doi:10.3389/fonc.2021.681717)
Supplement: Supplementary file 2 [file Table_2.docx]

**Supplementary Table S2|** List of the differential expression levels of 20 cross-regulatory genes in the radioresistant (RR) and invasion (Inv) sublines. The gene expression was determined by RT-qPCR analysis, with the relative fold changes in the sublines compared to their parental (Pt) cells. The clinical prognostic value was assessed by KM-Plotter suit analysis with the HNC patient cohort (n=500). For each gene, the P-value and Hazard Ratio (HR) were listed.

| **Assay**  **Gene** | **Gene expression** | | | | | | | | | **Prognosis** | |
| --- | --- | --- | --- | --- | --- | --- | --- | --- | --- | --- | --- |
|  | **OECM1 cells** | | | **Detroit cells** | | | **Average** | | | **KM Plot** | |
|  | **Pt** | **RR** | **Inv** | **Pt** | **RR** | **Inv** | **Pt** | **RR** | **Inv** | **P-value** | **HR** |
| AHNAK2 | 1 | 2.700 | 2.030 | 1 | 2.520 | 1.690 | 1 | 2.610 | 1.860 | 0.180 | 1.23 |
| BLMH | 1 | 0.796 | 1.616 | 1 | 1.131 | 1.179 | 1 | 0.963 | 1.397 | 0.025 | 0.74 |
| CEBPG | 1 | 1.632 | 0.905 | 1 | 1.535 | 0.540 | 1 | 1.584 | 0.723 | 0.048 | 1.40 |
| FLNB | 1 | 0.712 | 0.952 | 1 | 1.694 | 1.078 | 1 | 1.203 | 1.015 | 0.210 | 1.23 |
| GJA1 | 1 | 0.641 | 0.837 | 1 | 3.878 | 1.094 | 1 | 2.259 | 0.965 | 0.039 | 1.44 |
| GSK3B | 1 | 2.013 | 1.748 | 1 | 1.175 | 1.200 | 1 | 1.594 | 1.474 | 0.053 | 0.77 |
| HMOX1 | 1 | 4.864 | 2.020 | 1 | 27.695 | 1.931 | 1 | 16.279 | 1.976 | 0.018 | 0.70 |
| IGF1R | 1 | 1.453 | 0.925 | 1 | 1.388 | 1.267 | 1 | 1.420 | 1.096 | 0.025 | 1.37 |
| IL1R2 | 1 | 1.484 | 1.769 | 1 | 7.463 | 2.399 | 1 | 4.473 | 2.084 | 0.140 | 0.81 |
| IL6 | 1 | 14.275 | 2.332 | 1 | 2.094 | 0.861 | 1 | 8.184 | 1.597 | 0.0027 | 1.52 |
| ITGA6 | 1 | 3.750 | 4.860 | 1 | 7.980 | 4.770 | 1 | 5.865 | 4.815 | 2.80E-05 | 1.81 |
| LAMA3 | 1 | 2.180 | 1.043 | 1 | 0.180 | 2.201 | 1 | 1.180 | 1.622 | 0.110 | 1.26 |
| LAMC2 | 1 | 0.643 | 1.478 | 1 | 1.063 | 1.530 | 1 | 0.853 | 1.504 | 0.005 | 1.46 |
| MYH9 | 1 | 3.850 | 5.823 | 1 | 5.022 | 4.830 | 1 | 4.436 | 5.326 | 0.070 | 1.29 |
| MYL9 | 1 | 1.654 | 1.439 | 1 | 6.854 | 1.143 | 1 | 4.254 | 1.291 | 0.051 | 1.32 |
| NDRG1 | 1 | 1.734 | 1.264 | 1 | 10.855 | 3.039 | 1 | 6.295 | 2.151 | 0.027 | 1.37 |
| SerpinB2 | 1 | 2.067 | 1.664 | 1 | 0.434 | 5.261 | 1 | 1.250 | 3.462 | 0.035 | 0.74 |
| SLC1A1 | 1 | 0.498 | 2.192 | 1 | 1.205 | 0.393 | 1 | 0.851 | 1.293 | 0.150 | 0.81 |
| TGFB1 | 1 | 16.204 | 7.974 | 1 | 1.404 | 1.598 | 1 | 8.804 | 4.786 | 2.10E-04 | 1.95 |
| UBE2L3 | 1 | 1.043 | 1.189 | 1 | 1.566 | 1.082 | 1 | 1.305 | 1.135 | 0.0079 | 1.48 |
