# Supplementary material for: Molecular Interplays Between Cell Invasion and Radioresistance That Lead to Poor Prognosis in Head-Neck Cancer
Source: Front Oncol. 2021 Jul 9;11:681717. doi: 10.3389/fonc.2021.681717 (PMC8299304; doi:10.3389/fonc.2021.681717)
Supplement: Supplementary file 3 [file Table_3.docx]

**Supplementary Table S3|** List of the primers used in the present study.

| **Gene** | **Primer Sequences (5’ -> 3’)** |
| --- | --- |
| AHNAK2 | F: CCAAGTACTCGCAGGAAAGC |
|  | R: ATCTGCCTCTGGGAGCTGTA |
| BLMH | F: ATCAGAAAATTGGCCCCATAACA |
|  | R: TCTCCCTCCAACCATATTGCTT |
| CEBPG | F: CAAAAAGAGTTCGCCCAT |
|  | R: TGCAGTGTGTCTTGTGCTTTC |
| FLNB | F: TTGAATGGCGCAAAGGC |
|  | R: GCCCACCGTATTTGACAC |
| GJA1 | F: TGGTAAGGTGAAAATGCGAGG |
|  | R: GCACTCAAGCTGAATCCATAGAT |
| GSK3B | F: AGACGCTCCCTGTGATTTATGT |
|  | R: CCGATGGCAGATTCCAAAGG |
| HMOX1 | F: AAGACTGCGTTCCTGCTCAAC |
|  | R: AAAGCCCTACAGCAACTGTCG |
| IGF1R | F: AGGCTGAATACCGCAAAG |
|  | R: GTGAAAGGCCGAAGGTTAGA |
| IL1R2 | F: TCCTGCCGTTCATCTCATACC |
|  | R: CATCGTGTACGAGTAAGTGAGTG |
| IL6 | F: TCTGCGCAGCTTTAAGGAGT |
|  | R: ACAACAATCTGAGGTGCCCA |
| ITGA6 | F: TTGGAGCTTTTGTGATGGGC |
|  | R: GCTCAGTCTCTCCACCAACT |
| LAMA3 | F: CACCGGGATATTTCGGGAATC |
|  | R: AGCTGTCGCAATCATCACATT |
| LAMC2 | F: GCCTTTTGGCACCTGTATTC |
|  | R: CAGGATTCTCATCCCCTGAA |
| MYH9 | F: GAGCAAATGGGCCTGCT |
|  | R: TGTTGTCGGGCATGGA |
| MYL9 | F: ACCCCACAGACGAATACCTG |
|  | R: CCGGTACATCTCGTCCACTT |
| NDRG1 | F: GCAGAGTAACGTGGAAGTGGTC |
|  | R: CTCCACCACGGCATCCACT |
| SerpinB2 | F: AAATGGGCTTTATCCTTTCCGT |
|  | R: AGCTTTTCACGCAAGTACATCA |
| SLC1A1 | F: GCGAGGAAAGGATGCGAGT |
|  | R: GCTGTGTTCTCGAACCAAGACT |
| TGFB1 | F: GCCTCTGTGGCTCCTGCAATAAAC |
|  | R: CTTCTCGAAGAAGCTGCTGCCTC |
| UBE2L3 | F: TCGGGCTGACCTAGCTGAA |
|  | R: GGTCGCTTTTCCCCATATTTCT |
| GAPDH | F: GGAGCGAGATCCCTCCAAAAT |
|  | R: GGCTGTTGTCATACTTCTCATGG |
