# Supplementary material for: Molecular Interplays Between Cell Invasion and Radioresistance That Lead to Poor Prognosis in Head-Neck Cancer
Source: Front Oncol. 2021 Jul 9;11:681717. doi: 10.3389/fonc.2021.681717 (PMC8299304; doi:10.3389/fonc.2021.681717)
Supplement: Supplementary file 4 [file Table_4.docx]

**Supplementary Table S4|** Clinical characteristics of TCGA-HNSC patients with survival analysis.

**Parameter Number (%)**

**Gender**

Male 367 (73.4%)

Female 133 (26.6%)

**Age**

21 - 40 years old 20 (4.0%)

41 - 60 years old 228 (45.6%)

61 - 80 years old 229 (45.8%)

> 81 years old 23 (4.6%)

**Race**

Caucasian 426 (85.2%)

Asian American 10 (2.0%)

African American 47 (9.4%)

Others 17 (3.4%)

**Clinical stage**

Stg- I 27 (5.4%)

Stg- II 71 (14.2%)

Stg- III 81 (15.6%)

Stg- IV 264 (52.8%)

Others/Not defined 57 (11.4%)

**Total**  500 (100%)
